# The Mind-Writing Pupil: A Human-Computer Interface Based on Decoding of Covert Attention through Pupillometry

**DOI:** 10.1371/journal.pone.0148805

**Published:** 2016-02-05

**Authors:** Sebastiaan Mathôt, Jean-Baptiste Melmi, Lotje van der Linden, Stefan Van der Stigchel

**Affiliations:** 1 Aix-Marseille University, CNRS, LPC UMR 7290, Marseille, France; 2 Dept. of Experimental Psychology, Helmholtz Institute, Utrecht University, Utrecht, The Netherlands; University of Groningen, NETHERLANDS

## Abstract

We present a new human-computer interface that is based on decoding of attention through pupillometry. Our method builds on the recent finding that covert visual attention affects the pupillary light response: Your pupil constricts when you covertly (without looking at it) attend to a bright, compared to a dark, stimulus. In our method, participants covertly attend to one of several letters with oscillating brightness. Pupil size reflects the brightness of the selected letter, which allows us–with high accuracy and in real time–to determine which letter the participant intends to select. The performance of our method is comparable to the best covert-attention brain-computer interfaces to date, and has several advantages: no movement other than pupil-size change is required; no physical contact is required (i.e. no electrodes); it is easy to use; and it is reliable. Potential applications include: communication with totally locked-in patients, training of sustained attention, and ultra-secure password input.

## Introduction

A brain-computer interface (BCI) translates thought into action. BCIs provide new ways to interact with computers; importantly, they can restore the power to act and communicate in locked-in patients with little or no motor control [1,2].

There are many types of BCIs [3,4], which differ in the neural signal that they use (e.g., neural spikes or electroencephalography [EEG]), the way that neural activity is processed (e.g., through a classifier or by measuring overall activity in specific brain areas), and the actions that they perform (e.g. controlling a robotic limb, or writing text). Here we present a new method, which uses pupil size, rather than brain activity, as the controlling signal. Our method is related to two existing methods: the P300 speller [5], which is functionally similar to our method but relies on a different controlling signal; and a recent pupillometry-based method [6], which is functionally different from our method but relies on the same controlling signal.

P300 spellers are among the most successful BCIs [7]. They exploit the fact that rare visual stimuli elicit positive deflections in the EEG signal about 300 ms after their appearance. This event-related-potential (ERP) component is called the P300, and is largest for stimuli that are overtly (while looking at them) or covertly (without looking at them) attended [8]. In a classic P300 speller, the participant sees a grid of letters. One letter, or sometimes one full column or row of letters, is highlighted at a time. A P300 is elicited each time that a letter is highlighted. The participant selects a letter by attending to it, usually by looking at it directly (i.e. overt attention), which leads to an increased P300 when that letter is highlighted. In its simplest form, the letter that, when highlighted, elicits the strongest P300 is selected; however, most P300 spellers now use sophisticated classification techniques, and pool information from multiple ERP components [9]. But the principle remains the same.

The first P300 spellers relied heavily on direct fixation: When participants did not move their eyes, but attended covertly to the letters, accuracy was only around 60%. Thus, four out of ten times the system selected another letter than the user had intended [8,10]. This was problematic for real-world applications, because BCIs are mostly useful if they work without any overt (eye) movement; otherwise, movement-based methods, such as eye trackers [11] or the famous cheek-movement system used by Stephen Hawking, are much more efficient. However, modern P300 spellers no longer require (eye) movement, and reach impressive accuracy based on covert attention alone [12–14]. For example, a recent system that uses sequentially presented stimuli reached 97.1% selection accuracy with 1.35 characters per minute [12]. Expressed as information-transfer rate (ITR), which is a common measure for evaluating BCI performance [15], this corresponds to 6.18 bits/min. (Invasive techniques, using electrodes that are implanted in the brain, reach far higher ITRs [16]; however, their use is limited because few people are willing to undergo brain surgery [1].)

However, P300 spellers have several practical disadvantages. First, they require high-quality EEG-recording equipment, which is expensive. Low-cost EEG systems are becoming available, but, for the moment, are less reliable than more expensive systems [17]. Second, EEG electrodes must be carefully applied to the head. This is a tedious procedure that must be regularly redone, because current EEG systems are not designed for permanent use, and recording quality degrades over time. Electrodes also cause physical discomfort. Third, most P300 spellers require a calibration phase during which a classifier is trained on a person’s EEG signature. Again, this is tedious, and must be redone regularly to avoid performance degradation. These are hurdles for real-world applications [1].

Recently, a very different method, based on pupillometry, was developed and tested with partly locked-in patients [6]. This method exploits the pupillary dilation (enlargement) that accompanies effortful mental activities, such as arithmetic [18]. Participants were first asked a yes/ no question, and then sequentially shown two response options (‘yes’ followed by ‘no’, or vice versa; each option was shown once). A calculation was shown together with each response option (e.g. ‘29 x 49’). Participants were instructed to perform the calculation only during the interval of the intended response. The selection algorithm was simple: The response that elicited the strongest pupillary dilation (i.e. when the participant was calculating) was selected. Healthy participants reached around 90% selection accuracy with around 3.6 selections per minute (ITR = 1.93 bits/min; ITR is low because yes/ no selections carry little information). Typical locked-in patients reached around 70% accuracy with around 2.7 selections per minute (ITR = 1.42 bits/min). This method is much less efficient than a P300 speller; but it requires only a pupillometer (e.g. a remote camera), and does not require extensive preparation or calibration. These are advantages for real-world applications.

Here we present an entirely new human-computer interface (HCI) that combines the performance of a P300 speller with the usability of pupillometry. Our system builds on the recent discovery that the pupil constricts (shrinks) when you covertly attend to a bright stimulus, compared to a dark stimulus [19–22]. That is, unlike traditionally assumed, you do not need to look directly at a bright stimulus to elicit a pupillary light response; a covert shift of attention is sufficient [23]. Our method exploits this by presenting multiple letters within circles that oscillate between brightness and darkness. The participant selects a letter by covertly attending to it, without making any overt (eye) movement. The size of the pupil oscillates along with the brightness of the attended letter. This allows us to determine, reliably and in real time, which stimulus the participant intends to select.

## Results

### Phases 1–3: Selecting a Predefined Stimulus

**Fig 1:**
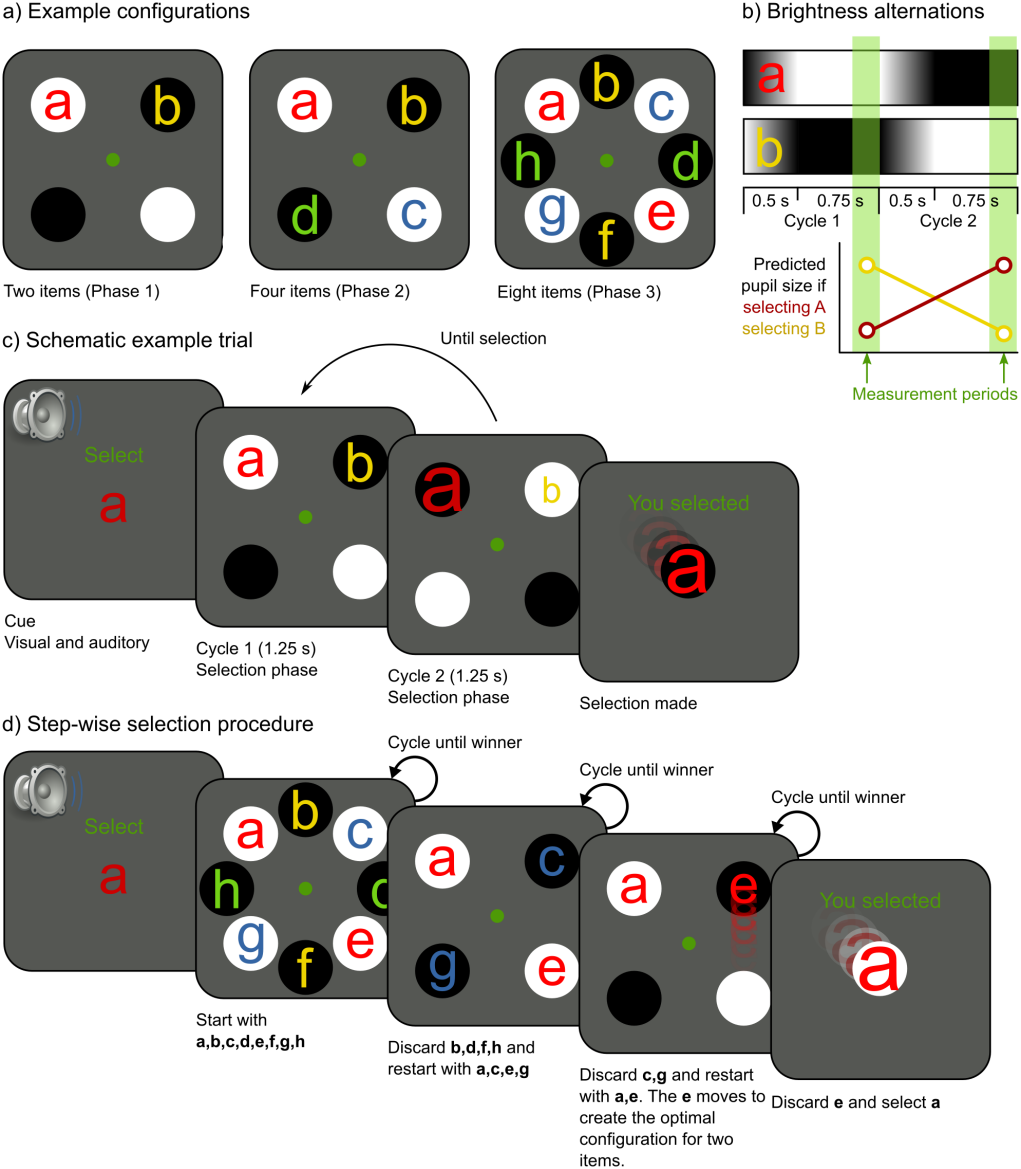
The selection procedure. a) Participants selected one of two (Phase 1), four (Phase 2), or eight (Phase 3) simultaneously presented stimuli. b) During each cycle, the brightness of the stimulus gradually changed in 0.5 s, and then remained constant for 0.75 s. Pupil size was measured during the last 0.25 s. c) The target stimulus was indicated by a cue. This example shows a correct selection, because the selected stimulus (‘a’) matches the cue. The size of the letters indicated how close they were to being selected. When a letter was selected, it smoothly moved toward the center. d) If there were more than two letters, letters were grouped by the brightness of their background. One group was eliminated on each selection, after which the remaining group was subdivided anew. This step-wise selection procedure repeated until a single winning stimulus remained.

In the first part of the experiment, participants learned to select one of two (Phase 1), four (Phase 2), or eight (Phase 3) letters (see Fig 1). Letters were presented within circles that oscillated between brightness and darkness in cycles of 1.25 s. Participants selected a letter by covertly attending to it, while keeping the eyes on the central fixation dot. We measured median pupil size during the last 0.25 s of each cycle, and used the following logic to determine which letter the participant intended to select: If pupil size decreased, the participant likely intended to select a letter that changed from darkness to brightness (‘b’ in Fig 1b); if pupil size increased, the participant likely intended to select a letter that changed from brightness to darkness (‘a’ in Fig 1b). The estimate of which letter the participant intended to select was updated after each cycle, until there was sufficient evidence for a reliable selection (Fig 1c); therefore, selection times varied. If there were more than two letters, letters were first divided into two groups, of which one was eliminated. This resulted in a step-wise selection procedure, in which eight letters were reduced to four, which were reduced to two, which were reduced to a single winner (Fig 1d). (For details, see Methods)

We designed the display to make selection as intuitive as possible. First, the size of the letters indicated how close they were to being selected; that is, a letter increased in size until it was selected. This type of sensory feedback is believed to increase BCI/ HCI performance [24]. Second, after a letter had been selected, it smoothly moved towards the display center. This animation increased the participants’ sensation of *grabbing* letters with their mind’s eye.

Training was considered successful if a participant reached at least 80% selection accuracy at the end of the training phase. This is more stringent than the threshold of 70% accuracy that is often taken as a lower limit for a useful BCI/ HCI [1,24].

#### Pupillary responses

**Fig 2:**
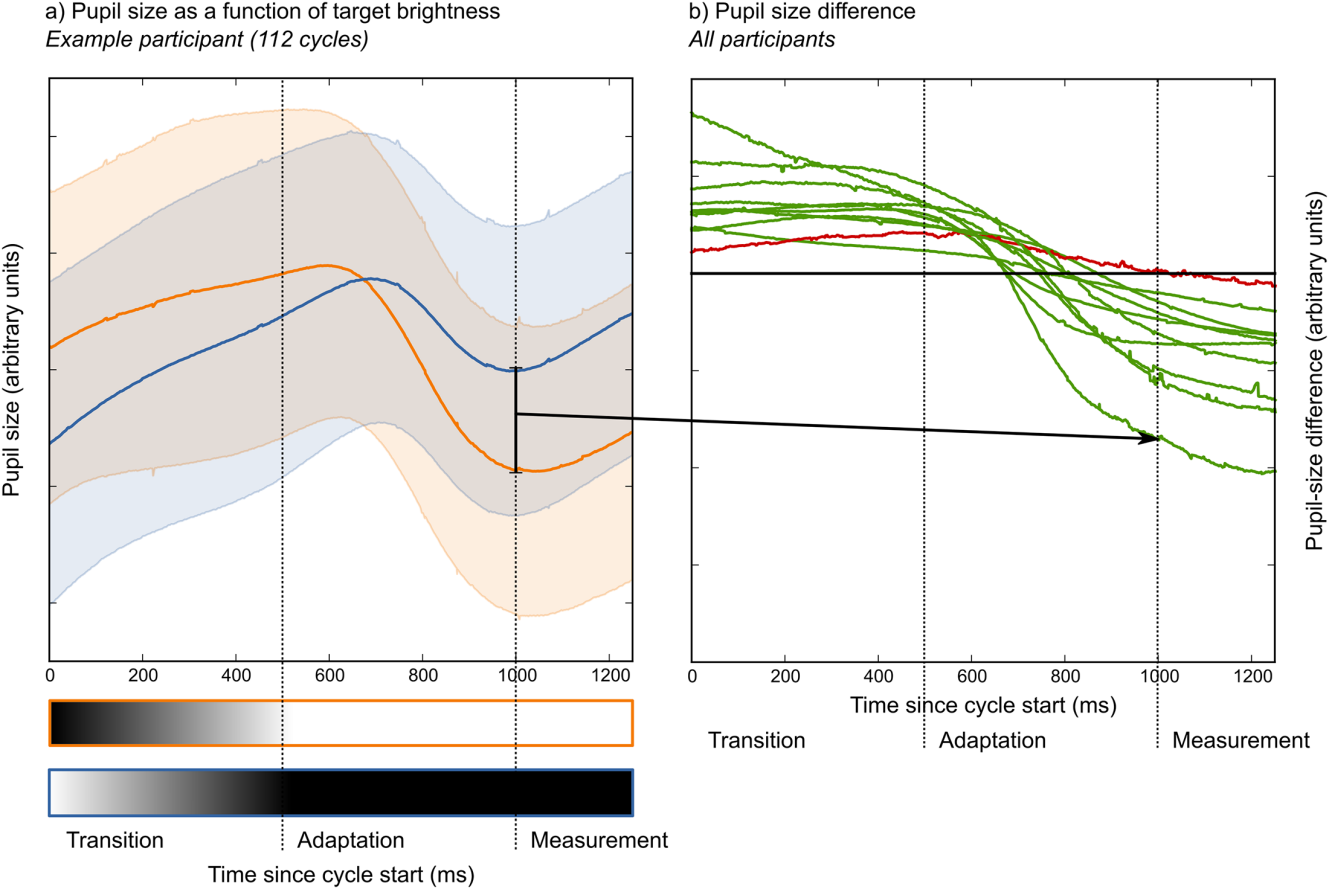
Pupillary responses during one brightness-transition cycle. a) Example data from one participant. Pupil size as a function of whether the target changes from bright to dark (blue line) or from dark to bright (orange line). Shadings indicate standard deviation. b) The pupil size difference (i.e. orange—blue) for all participants. The participant indicated in red did not reach our criteria for successful training. The participant indicated by the arrow corresponds to the example shown in (a). All data is from Phase 1, in which participants selected one out of two stimuli.

Fig 2a shows the average pupil size during a cycle, as a function of whether the attended stimulus changed from bright to dark (blue line) or dark to bright (orange line); this is based on the average of all cycles (*N* = 112) for a single participant during Phase 1. Each cycle started with a 0.5 s transition period, during which the brightness of the stimuli smoothly changed. During transition, pupil size still reflected the pretransition brightness: The pupil was larger if the attended stimulus was dark (orange line) than if it was bright (blue). Next, there was an adaptation period of 0.5 s. During adaptation, the pupil gradually started to reflect the new brightness of the attended stimulus, as reflected by the crossover of the blue and orange lines. Finally, there was a measurement period of 0.25 s, during which the brightness effect (i.e. the difference between the orange and blue lines) was roughly stable. Median pupil size during this period was used for the analysis; that is, our method exploited the fact that pupil size was larger when a target was dark (blue line) than when it was bright (orange line; see also Methods: Pupil-size measurement).

In addition to the effects of the brightness of the attended stimulus, there were also pronounced overall changes in pupil size during each cycle. Specifically, the brightness transition (0–0.5 s) induced a pupillary constriction around 0.2 s after the transition had finished; this is a pupillary response to visual change, which occurs even if overall luminance remains constant [25–27]. This constriction lasted only briefly, and was followed by a recovery (i.e. a redilation) that carried over into the start of the next cycle, resulting in an overall dilate-constrict-dilate pattern during each cycle.

As shown in Fig 2b, all participants showed a qualitatively identical pattern. One participant (indicated in red) showed a weak effect; this was the only participant who did not reach our criteria for successful training (see Results: Selection accuracy and speed).

#### Selection accuracy and speed

**Fig 3:**
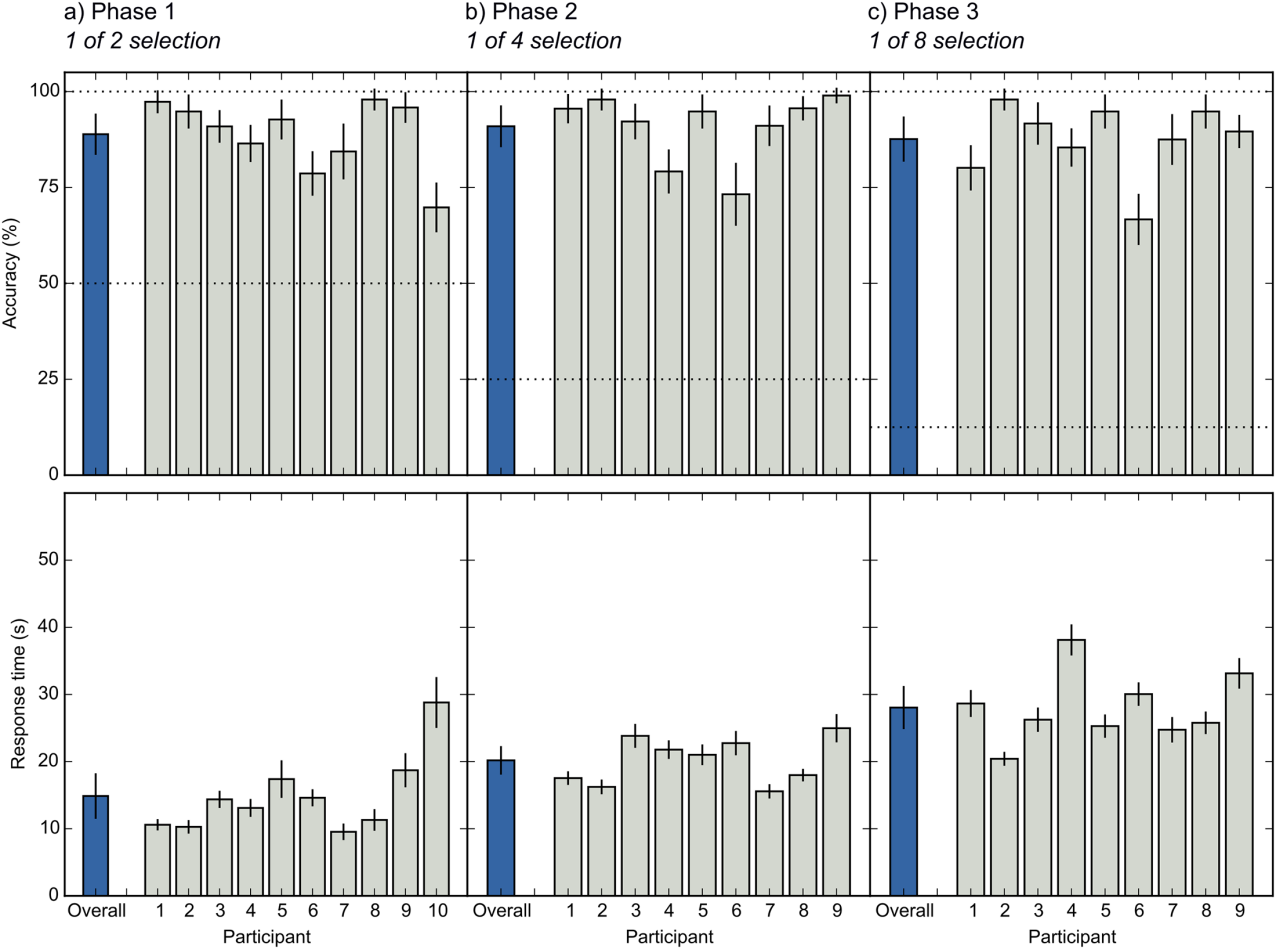
Selection accuracy (top row) and speed (bottom row) for individual participants (gray bars) and across participants (blue bars). Horizontal dashed lines indicate chance level. a) Results for Phase 1. b) Results for Phase 2. c) Results for Phase 3. Error bars indicate 95% confidence intervals, within-subject where applicable [28].

Fig 3 shows the mean selection accuracy and speed for each participant.

**Fig 4:**
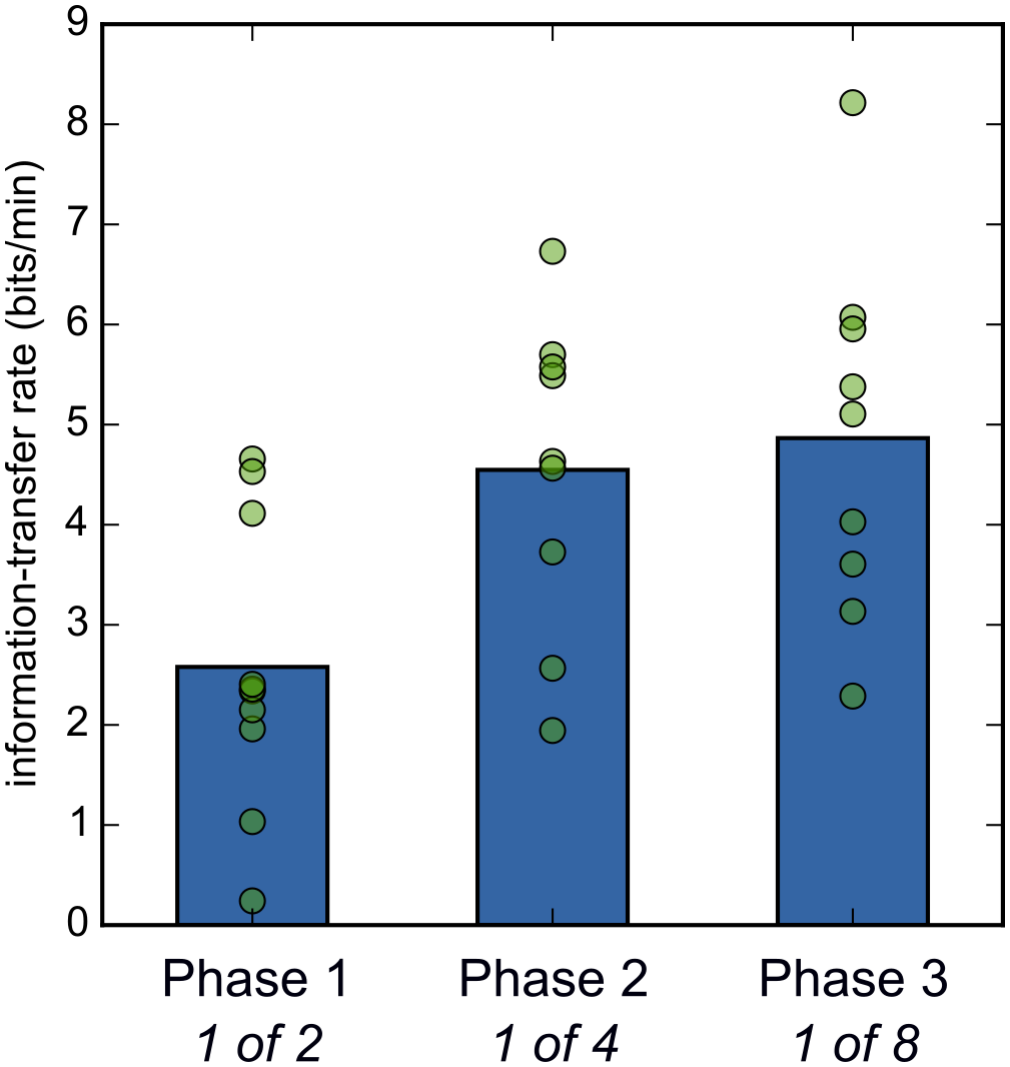
The information-transfer rate (ITR) in bits per minute. Bars indicate the mean ITR. Dots indicate individual participants.

In Phase 1, mean accuracy was 88.9% (chance = 50%; *N* = 10), with a mean selection time of 14.9 s. Information-transfer rate (ITR) was 2.58 bits/min (Fig 4; see Methods: Criteria and statistical analyses for a definition of ITR). Nine out of ten participants met our criteria for successful training (see Methods: Training program). One participant did not meet our predefined criteria for success, and therefore did not participate in subsequent phases (#10 in Fig 3; red line in Fig 2b); however, this participant’s accuracy was still 70%, which is often taken as the lower limit for useful HCI performance [1,24]. All other participants met our criteria for successful training (without increasing the decision threshold *T*; see Methods: Selection algorithm). In Phase 2, mean accuracy was 91.0% (chance = 25%; *N* = 9), with a mean selection time of 20.2 s. ITR was 4.55 bits/min. All participants met our criteria for successful training (without increasing *T*). In Phase 3, mean accuracy was 87.6% (chance = 12.5%; *N* = 9), with a mean selection time of 28.0 s. ITR was 4.86 bits/min. Again, all participants met our criteria for successful training (without increasing *T*).

#### Gaze independence

A crucial question is whether selection is fully independent of eye position. In each but the final block of each phase, the experiment was paused when fixation was lost (gaze deviated more than 2.6° from the display center for more than 10 ms), and continued when fixation was re-established. This controls for large eye movements, but not for small fixational eye movements. Therefore, in the final block of each phase, the entire display was locked to gaze position (from now on: gaze-stabilization mode): When the eyes drifted slightly to the left, all stimuli except the central fixation dot would shift slightly to the left as well. This made sure that selection was not driven by small eye movements in the direction of the attended stimulus [19,20].

**Fig 5:**
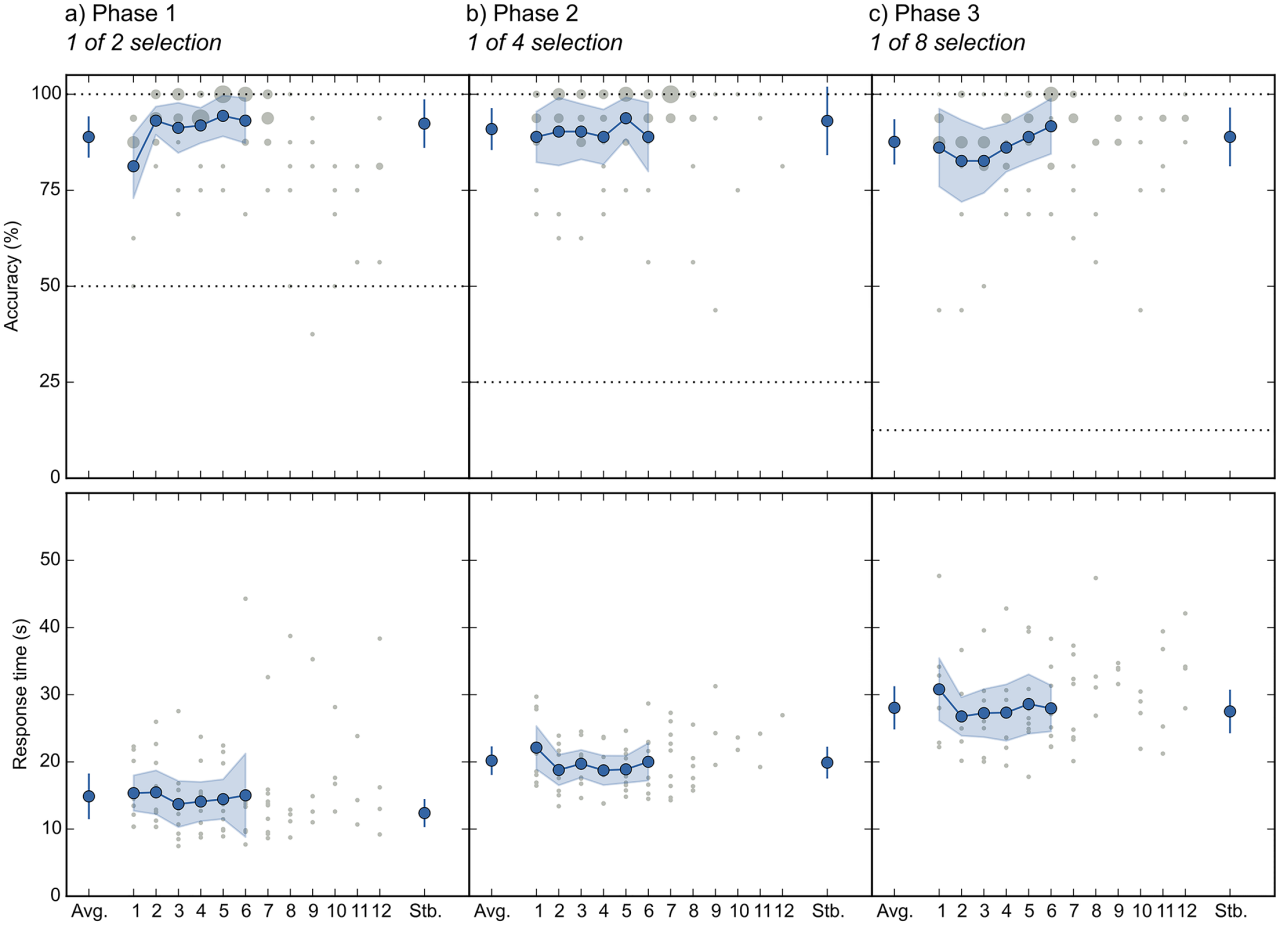
Selection accuracy (top row) and speed (bottom row) as a function of block number. Blue lines indicate across-participant means during the first six blocks, which were completed by all participants. The size of the gray circles indicates how often a score occurred. Performance during gaze-stabilization blocks is indicated by Stb. Horizontal dotted lines indicate chance level. a) Results for Phase 1. b) Results for Phase 2. c) Results for Phase 3. Error bars indicate 95% within-subject confidence intervals [28].

To test whether selection was independent of gaze, we conducted a Generalized Linear Mixed-Effects Model (GLMER) on accuracy with gaze stabilization (on/ off) as fixed effect (for details of statistical models, see Methods: Criteria and statistical analyses). This revealed no notable effect of gaze stabilization (*z* = 1.64, *p* = .102). A Linear Mixed-Effects Model (LMER) on response times also revealed no effect (*t* = 1.39, *p* = .174). If anything, performance was slightly better when gaze-stabilization mode was enabled (see also Fig 5 in which gaze-stabilization blocks are marked as ‘Stb.’).

Crucially, this shows that selection did not depend on small eye movements toward the attended stimuli [29], which participants could have made when gaze-stabilization was disabled. Our method is fully driven by covert attention.

#### Learning

Fig 5 shows how selection accuracy and speed evolved over time. To test whether significant learning occurred, we conducted a GLMER on accuracy with block (continuous) as fixed effect. This was done for each phase separately. There was no notable effect of block (i.e. no learning effect) in any phase: Phase 1 (*z* = 1.62, *p* = .104), Phase 2 (*z* = 1.30, *p* = .195), Phase 3 (*z* = 1.48, *p* = .139). An LMER on response time also did not reveal any notable effect of block: Phase 1 (*t* = 0.57, *p* = .565; intercept-only model), Phase 2 (*t* = 0.37, *p* = .721), Phase 3 (*t* = 0.73, *p* = .488).

Looking at Fig 5a, some learning did appear to occur between blocks 1 and 2 of Phase 1; that is, participants needed a single block of training, before they reached a more-or-less stable level of performance. Importantly, learning effects, if any, were small, and participants were able to use our method right away.

### Phase 4: Free Writing

**Fig 6:**
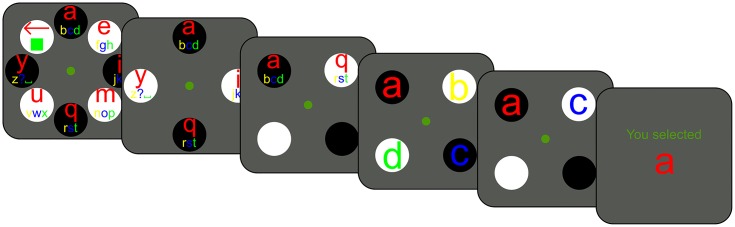
The symbol-selection procedure used for free writing. Initially, there are eight groups of characters and control symbols (‘backspace’, ‘space’, and ‘accept’). When one group has been selected (here ‘abcd’), it unfolds into four individual symbols (here ‘a’, ‘b’, ‘c’, and ‘d’), after which a final selection is made (here ‘a’).

In the final part of the experiment, participants used a virtual keyboard to write a self-selected sentence. This keyboard was similar to the displays used in Phases 1–3, but contained a full alphabet and several control symbols (see Fig 6). (For details, see Methods: Training program: Phase 4: Free writing.).

Eight out of nine participants successfully wrote a self-selected sentence (Table 1). The remaining participant wrote a sentence that was correct except for one typo.

**Table 1:** Results of Phase 4.

Response	Translation	Correct
LE CHAT DORT	The cat sleeps	Yes
JE NE SUIS PAS SI RAPIDE QUE CA	I’m not as fast as that	Yes
JE M APELLE ******	My name is ******	Yes
ENFIN TERMINEE	Finally finished	Yes
EXPERIENCE TERMINEE	Experiment finished	Yes
JE VAIS AGRANDIR	I’m going to get bigger	Yes
LE CHIEN BOIT	The dog drinks	Yes
VIVE LE POIIL?	Long live the fur?	Should have been “VIVE LE POIL?”
JE SUIS ****	I am ****	Yes

Results of Phase 4, during which participants wrote a self-selected sentence. Names have been replaced by asterisks (*).

Participants used a ‘backspace’ symbol to correct mistakes, and entered an ‘accept’ symbol to end text input. Therefore, we can distinguish the symbols that were entered (including characters that were later deleted, etc.) from the useful text (the text string that was eventually accepted). In total, participants entered 190 symbols (letters, ‘?’, ‘space’, ‘backspace’, and ‘accept’) for 133 characters of useful text (letters, ‘?’, and ‘space’). On average, one symbol took 51.1 s (*SD* = 9.6; including ‘backspace’ and ‘accept’), and one character of functional text took 75.2 s (*SD* = 20.5). The functional ITR was 3.91 bits/min. (A bug in an early version of the software occasionally required participants to enter unnecessary ‘backspace’ symbols. One sentence was affected by this issue, and was excluded from the analysis above.).

## Discussion

We have introduced a new human-computer interface (HCI) that is based on decoding of covert attention through pupillometry. Participants select a letter by covertly attending to it, without making any overt (eye) movement. Letters are presented within circles of oscillating brightness. Small changes in pupil size reflect the brightness changes of the attended stimulus [23], and this allows us to determine which stimulus the participant intends to select–in real time, independent of movement (other than pupil-size changes), and without physical contact.

In the experiment reported here, with healthy untrained participants, our method reached a selection accuracy of around 90%, and an information-transfer rate (ITR) of 4.86 bits/min (Fig 4). Out of ten participants, all but one reached our predetermined criteria for successful training; these criteria were exceptionally stringent, and even the unsuccessful participant achieved 70% selection accuracy, which is often taken as sufficient for a useful BCI/ HCI [1,24]. During pilot studies with highly trained participants (authors SM and LvdL), we have systematically reached near-perfect selection and ITRs of around 10 bits/min (see S1 Appendix). For comparison, P300 spellers that are based on covert attention (i.e. without eye movements) reach an ITR of around 6 bits/min [14], usually with a combination of trained and untrained participants [12,13]. The performance of our method is thus in the same range as that of the best noninvasive covert-attention BCIs to date.

Although all participants were able to select letters well above chance, there were considerable individual differences in selection speed and accuracy (see e.g. Fig 4). What drives these differences? At the moment, we can only speculate, but several factors are likely important. First, people differ in their ability and willingness to covertly attend to something for a long time, and to avoid distraction. Second, different people may use different strategies; for example, some participants reported to have visualized bright and dark things, or mentally rehearsed the words ‘bright’ and ‘dark’, in synchrony with the brightness transitions of the stimuli. This strategy of combining attention with mental imagery may have increased pupillary responses [30], thus improving selection performance. Finally, there are individual differences in the basic properties of the pupil: resting-state pupil size; how much the size of the pupil can change; and the amount of random pupil-size fluctuations [31]. It will be important to understand these factors to improve the efficacy of our system as a communication device.

An important advantage of our method, especially when compared to EEG-based methods, is its ease of use. Only a pupillometer, or an eye tracker that records pupil size, is required. For most experiments, we have used a research-grade eye tracker; but we have also successfully used an EyeTribe (The Eye Tribe Aps, Copenhagen, Denmark), a low-cost eye tracker that provides high-quality pupil-size measurements [32]. Our method does not require eye-position calibration, nor training of the selection algorithm. Together, these characteristics set our method apart from currently available methods.

An important application of an HCI/ BCI is as a communication channel for completely locked-in patients, that is, patients with complete loss of motor control [1]. P300 spellers and pupillometry-based methods have been tested successfully in partly locked-in patients with some remaining motor control [6,33]. But success with real-world applications has been modest, especially with completely locked-in patients. Important reasons for this limited success are [1]: difficulty of use (some methods require extensive training); low selection accuracy; skin problems due to EEG electrodes; low selection speeds; and the need for sustained attention. Our method solves some of these problems by providing ease of use, avoiding physical contact, and providing high selection accuracy. But other challenges remain, notably low selection speed and the need for sustained attention. In addition, it is unclear to what extent the pupillary light response, which our method relies on, remains intact in completely locked-in state [34]. Therefore, future studies are needed to determine how well our method, or a variation thereof, works in patient groups.

A second application of our method is as an ultra-secure way to enter passwords or PIN codes. Imagine a cash machine that is equipped with a pupillometer. To enter a PIN code, the user would be shown a display similar to that depicted in Fig 1a, and enter digits by covertly attending to them. Based on our results (see Fig 3), entering a four-digit PIN code would take around two minutes. This is slow, but feasible, and could be useful in situations that require high security.

A third application of our method is as a way to train sustained attention. To select a letter, participants must attend to it for some time, which is effortful. Therefore, a game-like variation of our method could be an attractive way to train sustained attention. The main benefit of our method over regular attention-training exercises is direct feedback: The user can be immediately notified when there is a lapse of attention. (In our experiments, feedback was provided by changing the size of the target letter.)

In conclusion, we have presented a new pupillometry-based method to translate thought into letters. Our method is highly accurate and easy to use, and does not require elaborate equipment, preparation, or training. We have highlighted communication with completely locked-in patients, ultra-secure password input, and training of sustained attention as possible applications.

## Methods

### Preregistration

This experiment was preregistered on Jan 21, 2015 (https://osf.io/yvaqs/). Whenever a deviation from registration occurred, it is indicated in the sections below.

### Materials and Availability

Participant data, experimental software, and analysis scripts are available from: https://github.com/smathot/mind-writing-pupil. This repository also includes a ready-to-use package for using our HCI with supported systems (currently tested with EyeLink and EyeTribe eye trackers, and Windows and Linux operating systems). A screencast of our method is available on-line: https://youtu.be/cGfkD2opTz4

### Participants

Ten naive participants from the community of Aix-Marseille Université were recruited (normal or corrected vision; 7 women; age range: 20–25). Participants received €90 for their participation (deviation from preregistration: We originally planned to pay €60). Participants provided written informed consent prior to the experiment. The study was conducted with approval of the ethics committee of Aix-Marseille Université (Ref.: 2014-12-03-09), and conformed to the Declaration of Helsinki (7^th^ rev.).

### Software and Apparatus

Eye position and pupil size were recorded monocularly with an EyeLink 1000 (SR Research, Mississauga, ON, Canada), a video-based eye tracker sampling at 1000 Hz. The right eye was recorded, unless the left eye provided a better signal. Stimuli were presented on a 21" ViewSonic p227f CRT monitor (1280 x 1024 px, 85 Hz) running Ubuntu Linux 14.04. Testing took place in a dimly lit room. The experiment was implemented with OpenSesame [35] using the PsychoPy back-end [36] for display control and PyGaze [37] for eye tracking.

### General Stimuli and Procedure

Before each block, a nine-point eye-tracker calibration was performed. At the start of each selection trial, an automatic single-point recalibration (drift correction) was performed. The display consisted of a green central fixation dot (*r* = 0.2°) on a gray background (13.0 cd/m^2^). Items were presented in a circular configuration at an eccentricity of 9.2° (Fig 1). Items consisted of colored letters against a circular background (*r* = 3.1°). When only two items were presented, each item was accompanied by a mirror-symmetric placeholder (see Fig 1a; this configuration was chosen because pilot experiments showed it to be the most effective of several tested configurations; see S1 Appendix). The backgrounds alternated between brightness (97.0 cd/m^2^) and darkness (5.1 cd/m^2^) in cycles of 1.25 s (0.8 Hz). Each cycle consisted of a smooth brightness transition of 0.5 s, followed by 0.75 s of constant brightness (Fig 1b).

The participant attended covertly to the target stimulus, while keeping gaze on the central fixation dot. The target was either indicated by a cue (Phase 1–3) or chosen by the participant (Phase 4). The cue was both visual (e.g., the letter ‘A’ shown on the display) and auditory (e.g., a synthesized French voice saying *Sélectionnez A*). The participant could replay the auditory cue at any moment by pressing the space bar. The trial ended when a selection was made (Fig 1c, see Selection algorithm).

### Selection Algorithm

Letters are divided into two groups: bright and dark backgrounds. Each group has a parameter *L* that reflects how likely it is that the attended letter is part of that group. Initially, *L* is 1 for both groups. After each cycle, a proportional pupil-size difference (*PPSD*) is determined (see Pupil-size measurement). For the letter group that has changed from bright to dark, *L* is multiplied by *PPSD* (because we expect the pupil to dilate if the target is part that group). For the letter group that has changed from dark to bright, *L* is divided by *PPSD* (because we expect the pupil to constrict if the target is part that group). Cycling continues until the proportional difference between the *L*s for both groups exceeds a threshold *T* (*L1*/*L2* > *T* or *L1/L2* < 1/*T*), after which the group with the highest *L* is designated as the winner. If groups consist of more than one letter, the losing group is discarded, and the winning group is subdivided into two new bright/ dark groups (See Fig 1d). The selection process then starts anew. This continues until the winning group contains only a single letter, after which the final selection is made. The analysis is performed on-line, while the participant performs the task.

A crucial property of this algorithm is that it continues until there is sufficient evidence for reliable selection. Selection can be made faster but less accurate by reducing the threshold *T*, and slower but more accurate by increasing it.

The reason that we presented up to eight separate letters, even though the algorithm made only one-of-two selections, was to avoid users from having to re-orient their attention after each selection; that is, once users shifted their attention toward a to-be-selected letter, they simply kept attending to it, while the algorithm gradually pruned the non-attended letters through a series of one-of-two selections.

### Pupil-Size Measurement

The proportional pupil-size difference on cycle *i* (*PPSD(i)*) is defined as:

$$PPSD\left(i\right)=\frac{PS\left(i\right)}{PS\left(i-1\right)}$$

Here, *PS(i)* is the median pupil size during the last 250 ms of cycle *i* (see Fig 1b).

### Training Program

The training program consisted of four phases. In Phases 1–3, participants were trained to make progressively more complicated selections. In Phase 4, participants wrote a short self-selected sentence using an extension of the technique trained in Phases 1–3. Training took about 10 hours, spread over multiple days.

#### Phases 1–3: Selecting a predefined stimulus

In Phase 1, participants were trained to select one of two simultaneously presented stimuli. Blocks consisted of 16 selections.

Training was successful when participants reached: 100% accuracy after completing at least 6 blocks; or at least 80% accuracy on block 12. Thus, participants completed between 6 and 12 blocks. If training was unsuccessful, the phase was restarted with a more conservative threshold of 1.5 (default threshold = 1.375). If training then failed again, the experiment was aborted and training was considered unsuccessful for that participant. After training was successfully completed, participants completed a single block in gaze-stabilization mode. Our criteria for success were stringent: Commonly, 70% accuracy is taken as a lower limit for a useful BCI/ HCI [1,24].

Phases 2 and 3 were identical to Phase 1, except that participants selected one out of four (Phase 2) or eight (Phase 3) stimuli.

#### Phase 4: Free writing

In Phase 4, participants trained to write text by selecting characters and control symbols (‘backspace’: a leftward arrow; ‘space’: a low bar; and ‘accept’: a square) on a virtual keyboard. The participant initially selected one of eight symbol groups. This group then unfolded, after which the participant selected one symbol. Structurally, selecting a symbol was therefore identical to a one-of-eight selection (Phase 3) followed by a one-of-four selection (Phase 2), or, in the case of ‘accept’ and ‘backspace’, a one-of-two selection (Phase 1). This procedure is similar to the Hex-o-Spell P300-based human-computer interface [38].

First, participants were given a print-out of the virtual keyboard to familiarize themselves with its layout (see Fig 6). Next, they practiced by writing the French word “ecrire” (without accent). Practice was completed when the word was written successfully, with a maximum of three attempts. Next, participants chose a short sentence (deviation from preregistration: several participants wanted to write a long sentence, and we therefore abandoned our initial maximum of 15 characters). Participants were given two opportunities to write this sentence. Writing was considered successful when the final sentence matched the specified sentence. The use of backspace to correct mistakes during text input was allowed.

#### Criteria and statistical analyses

No participants or selections were excluded from the analysis. Two blocks (32 selections) were lost due to technical problems. Two participants chose not to finish the experiment, and were replaced. In total, 257 blocks (4,112 selections) were included in the analysis.

We analyzed accuracy using Generalized Linear Mixed-Effects Models with correctness (binomial) as dependent variable. We analyzed response times using Linear Mixed-Effects Models with response time as dependent variable. We included by-participant random intercepts and slopes (i.e. maximal random effects), unless this model failed to converge, in which case we included only random intercepts. Fixed effects were considered reliable when *p* < .05; however, we emphasize general patterns over significance of individual results. These analyses were conducted in R [39], using the packages lme4 [40] and lmerTest [41].

Information-transfer rate (ITR) is a measure of communication efficiency, and depends on both accuracy and speed. ITR was determined using the following formula [15]:

$$ITR=\frac{log_{2}N+Acclog_{2}Acc+\left(1-Acc\right)log_{2}\frac{1-Acc}{N-1}}{RT/60}$$

Here, *ITR* is in bits per minute, *N* is the number of response options, *Acc* is proportion correct responses, and *RT* is the response time in seconds.

The response time was the interval between the start of the first selection cycle and the end of the last selection cycle. Mean accuracy, response time, and ITR were first determined per participant, and then averaged to arrive at grand means (i.e. a means-of-means approach).

## Supporting Information

S1 Appendix Description of pilot experiments. Prior to the training program discussed in the main text, we conducted five pilot experiments to optimize the system’s design.
(PDF)
