# Supplementary material for: The Mind-Writing Pupil: A Human-Computer Interface Based on Decoding of Covert Attention through Pupillometry
Source: PLoS One. 2016 Feb 5;11(2):e0148805. doi: 10.1371/journal.pone.0148805 (PMC4743834; doi:10.1371/journal.pone.0148805)
Supplement: S1 Appendix — Prior to the training program discussed in the main text, we conducted five pilot experiments to optimize the system’s design. (PDF) [file pone.0148805.s001.pdf]

# The Mind-Writing Pupil: A Human-Computer Interface Based on Decoding of Covert Attention through Pupillometry (S1 Appendix)

Sebastiaan Mathôt[1]*, Jean-Baptiste Melmi[1], Lotje van der Linden[1], and Stefan Van der Stigchel[2]

[1] Aix-Marseille University, CNRS, LPC UMR 7290, Marseille, France

[2] Dept. of Experimental Psychology, Helmholtz Institute, Utrecht University, Utrecht, The Netherlands

* Corresponding author

Address for correspondence:

Sebastiaan Mathôt, PhD

Aix-Marseille Université / CNRS

Laboratoire de Psychologie Cognitive

3 Place Victor Hugo

Centre St. Charles, Bâtiment 9, Case D

13331 Marseille

France

s.mathot@cogsci.nl

http://www.cogsci.nl/smathot

Prior to the training program discussed in the main text, we conducted five pilot experiments to optimize the system's design. In all pilot experiments, two authors (SM and LvdL) served as participants. (The authors only participated in the pilot experiments; for the training program described in the main text, all participants were naive.) In each experiment, we varied one parameter, and then continued with the parameter value that resulted in fastest responses. We tested only one-of-two selections. These pilot experiments were purely exploratory; no statistical testing was performed, and no interpretations are provided. We report these pilot experiments for completeness, but their main goal was to aid in the design of the system.

## Accuracy

Across all pilot experiments, only 5 of 2,064 selections were incorrect (99.8% accuracy). Therefore, accuracy was not analyzed further.

## Starting parameters

Aside from the parameters discussed below, the procedure and stimuli were identical to those described in the Methods of the main text. We started with a stimulus eccentricity of 7.3°, a stimulus size of 1.5° (i.e. the radius of the stimulus' backgrounds), and a display-background luminance of 22.9 cd/m².

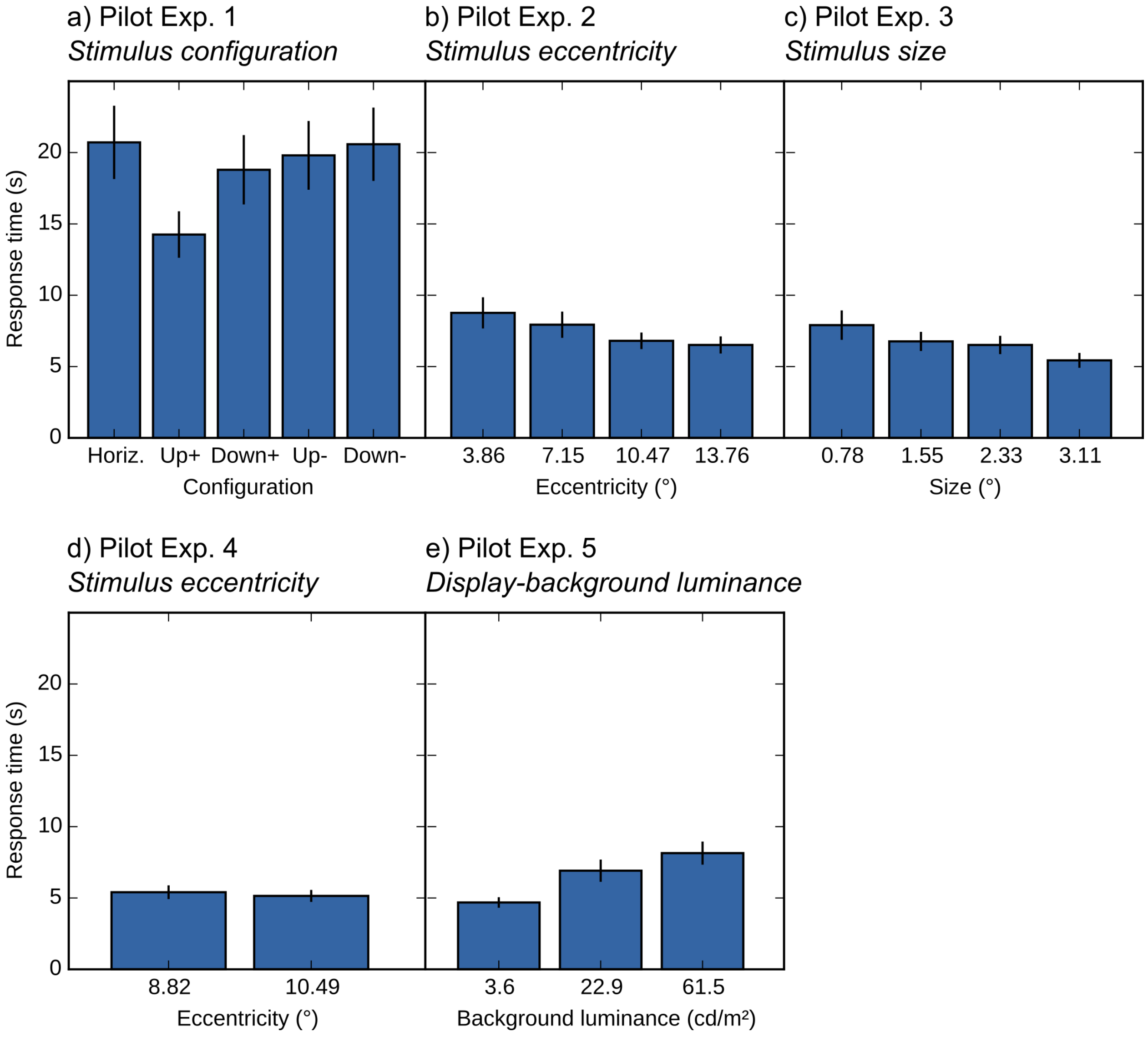


***Figure 1.*** *The results from the pilot experiments. Error bars indicate 95% confidence intervals (based on all trials, across both participants).*

## Pilot Experiment 1: Stimulus configuration

We tested the following geometric configurations (80 selections per configuration):

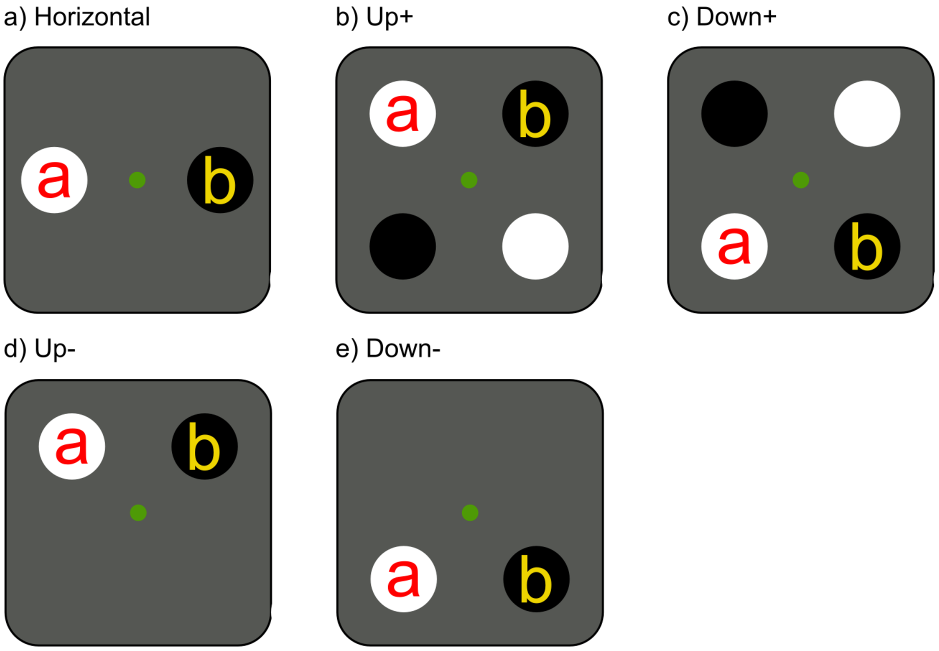


***Figure 2.*** *The five stimulus configurations that were tested in Pilot Experiment 1.*

- Horizontal: Two letters on the horizontal axis, left and right of fixation ( Figure 2a).
- Up+: Two letters in the upper visual field with mirror-symmetric placeholders in the lower visual field (Figure 2b).
- Down+: Two letters in the lower visual field with mirror-symmetric placeholders in the upper visual field (Figure 2c).
- Up-: Two letters in the upper visual field ( Figure 2d).
- Down-: Two letters in the lower visual field (Figure 2e).

Configuration was varied within blocks. Based on response time ( Figure 1a), we continued with the Up+ configuration.

### Pilot Experiment 2: Stimulus eccentricity

We tested the following stimulus eccentricities (128 selections per eccentricity): 3.86°, 7.15°, 10.47°, and 13.76°. Eccentricity was varied within blocks. Based on response time (Figure 1b), we continued with an eccentricity of 13.76°.

### Pilot Experiment 3: Stimulus size

We tested the following stimulus sizes (128 selections per size): 0.78°, 1.55°, 2.33°, and 3.11°.

The size referred to the radius of the stimulus' backgrounds. Size was varied within blocks. Based on response time (Figure 1c), we continued with a stimulus size of 3.11°.

### Pilot Experiment 4: More stimulus eccentricities

We felt that the optimal eccentricity might differ for the new stimulus size selected during Pilot Experiment 3 (3.11°). In addition, we noticed that a full circle of stimuli did not fit on the screen with the previously selected eccentricity (13.76°). Therefore, we tested two new eccentricities (128 selections per eccentricity): 8.82° and 10.49°. Eccentricity was varied within blocks.

There was little overall effect of eccentricity on selection speed ( Figure 1d), and the optimal eccentricity was different for the two participants (not shown). Therefore, we continued with an intermediate eccentricity of 9.2°.

### Pilot Experiment 5: Display-background luminance

We tested the following display-background luminances (128 selections per size): 3.6 cd/m² (dark), 22.9 cd/m² (medium), and 61.5 cd/m² (bright). Background luminance was varied between blocks of 16 selections. Block order was randomized.

Clearly, dark display backgrounds are more effective than bright display backgrounds (Figure 1e). However, the darkest display background that we tested (3.6 cd/m²) seemed to merge with the dark phase of the stimulus' backgrounds; that is, the stimulus' backgrounds seemed to disappear when they were dark. Therefore, we continued with a slightly less dark display background (13.0 cd/m²).

## Acknowledgements

The research leading to these results has received funding from the People Programme (Marie Curie Actions) of the European Union's Seventh Framework Programme (FP7/2007-2013) under REA grant agreement n° 622738 awarded to Sebastiaan Mathôt (http://cordis.europa.eu/project/rcn/187709_en.html). In addition, this research was funded by a VIDI Grant 452-13-008 from the Netherlands Organization for Scientific Research to Stefan Van der Stigchel (http://www.nwo.nl/en/research-and-results/programmes/Talent+Scheme/awards/vidi+awards/vidi+awards+2013). The funders had no role in study design, data collection and analysis, decision to publish, or preparation of the manuscript.

## Competing financial interests

The authors declare no competing financial interests.

## Author contributions

JBM collected the data. SM wrote the first draft of the manuscript. SM and LvdL performed pilot testing. All authors contributed to the design of the experiment, and revision of the manuscript.
